# Social and Emotional Learning in the Ibero-American Context: A Systematic Review

**DOI:** 10.3389/fpsyg.2021.738501

**Published:** 2021-09-30

**Authors:** Francisco-Domingo Fernández-Martín, José-María Romero-Rodríguez, José-Antonio Marín-Marín, Gerardo Gómez-García

**Affiliations:** ^1^Department of Developmental and Educational Psychology, Campus Universitario de Cartuja, Granada, Spain; ^2^Department of Didactics and Scholar Organization, Campus Universitario de Cartuja, Granada, Spain

**Keywords:** social and emotional learning (SEL), socioemotional competences, program assessment, primary education, secondary education

## Abstract

Social and emotional learning (SEL) has acquired great prominence in recent years, due to the skills it develops in students, influencing personal and social well-being. At the same time, society is moving toward a model in which understanding oneself and others is a fundamental aspect in order to function properly on a social level. Studies on SEL programmes have been carried out in various parts of the world, although recent reviews have focused exclusively on the Anglo-Saxon context. Therefore, the aim of this paper was to synthesize research on the efficacy and effectiveness of SEL programmes in Ibero-American contexts in early childhood, primary and secondary education. Systematic review was used as the method of enquiry, following the standards of The Campbell Collaboration. In total, 22 empirical studies of SEL programmes implemented in Ibero-America were collected. The results showed that the SEL variables with the highest incidence and significant results were self-awareness, social awareness, self-control, relationship skills, decision-making, school climate, well-being, and academic achievement. While no studies focused on sense of belonging or school safety. Finally, the establishment of programme components, duration, and integration, for each variable, scientifically evidences the keys that can ensure the success of future SEL programmes.

## Introduction

Over the past few years, there has been a considerable increase in educational, social, and political interest in social and emotional learning (SEL), a trend that seems to have arisen from the need to address the high incidence of social, emotional, and behavioral problems among children, adolescents and young adults in today's society, and to build protective factors that enhance their well-being and performance (Oberle et al., 2016). SEL is conceived as “the ability to coordinate cognition, affect, and behavior that enables people to thrive in diverse cultures and contexts and to achieve specific tasks and positive developmental outcomes” (Mahoney et al., 2020, p. 4), and commonly refers to the process in which individuals acquire and effectively apply the knowledge, skills and attitudes necessary to develop healthy identities, manage their emotions, set and achieve positive personal and collective goals, feel and show empathy for others, establish and maintain positive and supportive interpersonal relationships, manage interpersonal situations constructively, and make responsible and caring decisions (Payton et al., 2008; Durlak et al., 2011; Gutman and Schoon, 2013; Collaborative for Academic, Social, and Emotional Learning, 2015, 2020; Weissberg et al., 2015; Taylor et al., 2017; Jagers et al., 2019; Mahoney et al., 2020).

In this sense, SEL is an essential component for personal and socio-emotional development, essential for the learning and success of any person in the different domains of their lives, as it provides them with the necessary tools to effectively and efficiently face the various daily tasks and challenges, thus increasing their satisfaction and productivity (Weissberg et al., 2015; Oberle et al., 2016). To this end, SEL entails the design, implementation and evaluation of a coordinated set of evidence-based intervention programmes and practices that promote a range of social-emotional competencies, such as self-awareness, social awareness, self-control, relationship skills and responsible decision-making, among its participants by establishing safe and supportive learning environments (Collaborative for Academic, Social, and Emotional Learning, 2015, 2020; Jagers et al., 2019; National Commission on Social, Emotional, and Academic Development, 2019; Mahoney et al., 2020).

Indeed, numerous studies have established certain causal links between SEL programmes and certain socio-emotional, behavioral, and academic outcomes of their participants. Early systematic reviews aimed at determining the impact of these interventions (i.e., Diekstra, 2008; Payton et al., 2008; Durlak et al., 2010, 2011; Sklad et al., 2012) already confirmed the multiple benefits that SEL programmes generate among early childhood, primary and secondary school students, regardless of their socio-demographic and educational characteristics (i.e., children and adolescents from diverse racial, ethnic and socio-economic backgrounds, with and without emotional and behavioral problems, from different educational levels and settings). Programmes of this type, implemented and evaluated both in and out of school, have repeatedly demonstrated their ability to improve students' social-emotional skills, their self-perceptions, their attitudes toward others, their commitment and bond with the school, their prosocial behavior and their academic performance, also promoting a reduction in their emotional, behavioral, and substance abuse problems (Diekstra, 2008; Payton et al., 2008; Durlak et al., 2010, 2011; Sklad et al., 2012). Moreover, the variability of results among some of the interventions included in these initial reviews stimulated interest in identifying those elements and characteristics that most guarantee the success of these intervention measures, i.e., the presence of those practices recommended for the development of socioemotional skills in students, such as using a step-by-step sequenced training approach (sequenced), emphasizing active forms of learning for students to practice the new skills (active), concentrating specific time and attention on skills training (focused) and clearly defining goals (explicit) (SAFE) (Durlak et al., 2010, 2011). In fact, collaborative learning is one of the most frequently used active forms of learning for students to practice new skills (active) (Durlak et al., 2011). Collaborative learning experiences increase the effectiveness of SEL programmes, even more so when supported by technology (Stahl, 2002; González-González et al., 2014; Claros et al., 2016; Collazos et al., 2021).

The results of the other meta-analytic reviews that have been conducted on SEL programmes are along the same lines, including the work of Jagers et al. (2015); Wigelsworth et al. (2016); Sabey et al. (2017); Taylor et al. (2017); Corcoran et al. (2018); Yang et al. (2019), and Murano et al. (2020), not to mention the Collaborative for Academic, Social, and Emotional Learning (2015) guide, which selects and assesses selected SEL interventions, along with evidence on their effectiveness. Specifically, Jagers et al. (2015) found that participation in this type of programme promotes the development of socioemotional competencies among students, in addition to reducing the risk of social exclusion, in very similar terms to the results obtained by Wigelsworth et al. (2016) and Taylor et al. (2017), who also provided evidence on the importance of the type of evaluation to which these interventions are subjected, the people involved in it and the country in which they are implemented (Wigelsworth et al., 2016), as well as on the temporal stability of the results (Taylor et al., 2017). Analyses by Sabey et al. (2017) showed that SEL interventions that incorporate behavioral training have greater effects on the development of prosocial behavior and decreases in antisocial behavior compared to programmes that focus exclusively on social-emotional development. Corcoran et al. (2018) explored research on the effects of these intervention measures on performance in mathematics, reading and science, identifying a positive effect compared to results obtained through traditional methods. Finally, the analyses of the work carried out by Yang et al. (2019) showed that SEL programmes generate improvements in the socioemotional competence of students at risk of academic failure and social exclusion, while the results of Murano et al. (2020) highlight the impact of this type of intervention on the general development of students' socioemotional skills and the reduction of their behavioral problems.

These results have contributed to SEL interventions being among the most successful child and youth development programmes (Payton et al., 2008), which has led to their rapid and widespread diversification and incorporation into schools and classrooms around the world (Wigelsworth et al., 2016), even generating effects on other members of the educational community. For example, this is the case for teachers, among whom higher rates of effectiveness and achievement have been identified in their teacher planning compared to those who did not experience it (Domitrovich et al., 2007; Oberle et al., 2016). However, most of the meta-analytical evidence available on the efficacy and effectiveness of SEL programmes is derived from research carried out in the Anglo-Saxon context, or in which cross-cultural adaptations originally validated in this context have been evaluated, when various interventions based on the SEL model have been developed in Latin America, as is the case of the programmes “INTEMO,” “Programa curricular socioemocional,” “Educación Emocional,” “EDI program,” “Programa aulas felices,” “Siendo inteligente con la emociones,” or “Aprendiendo a ser” (Aguilar et al., 2019).

Taking into account these previous considerations, the purpose of this systematic review was to synthesize the research on the efficacy and effectiveness of SEL programmes in Ibero-American contexts of early childhood, primary and secondary education. Ibero-American context was established as geographical and/or cultural restriction because the available systematic reviews and meta-analyses on SEL programmes have focused mainly on research carried out in the Anglo-Saxon context, or those that include cross-cultural adaptations originally validated in this context (Wigelsworth et al., 2016), while early childhood, primary and secondary education were determined as participating population because these are the educational stages in which this type of programme is most developed and recommended, especially as a preventive measure against the problems of school adjustment that students tend to present (Taylor et al., 2017; Jones et al., 2019; Mahoney et al., 2020). The following specific objectives were posed: (a) identify the main characteristics of the research that has been carried out at the Ibero-American level on the efficacy and effectiveness of SEL programmes in early childhood, primary and secondary education; (b) describe the most relevant characteristics of SEL programmes implemented in Ibero-American contexts, as well as the empirical evidence of their efficacy and effectiveness in improving the academic and socio-emotional outcomes of students in pre-primary, primary, and secondary education; and (c) issue relevant conclusions and recommendations for future educational practices and policies in this field.

In addition, following the international standards set by The Campbell Collaboration (2019), the following research questions were posed:

1. What are the most outstanding characteristics of the studies (i.e., geographical and temporal distribution, publication typologies, sample selection procedures and group configuration, sample characteristics, evaluation instruments and methodological designs) that have been developed in Latin American countries on this type of intervention with pre-school, primary, and secondary school students?
2. What are the most relevant characteristics of the SEL programmes (i.e., environment in which they are developed, components, procedures, practices, strategies, techniques, and intervention resources) that have been implemented in Latin American contexts with students between 3 and 18 years of age?
3. What is the significant evidence of the studies, with respect to the variables of the SEL model, to generate socioemotional and academic improvements among infant, primary and secondary school pupils in the Ibero-American context?

## Methods

The first action of this systematic review, as specified in the guidelines set out by The Campbell Collaboration (2019) and the Preferred Reporting Items for Systematic Reviews and Meta-Analyses (PRISMA) statement (Page et al., 2021), was to design and plan a protocol for its development. The protocol can be found in Fernández-Martín et al. (2020).

### Inclusion and Exclusion Criteria

The inclusion criteria were determined according to the general objective of the systematic review, initially establishing the definition or operational characteristics of the independent and dependent variables, and then specifying the methodological designs, the participating population and the geographical, cultural and temporal restrictions.

In this sense, SEL programmes (independent variable) refer to those educational interventions that are based on the SEL model and are aimed at students acquiring and effectively applying the knowledge, skills, and attitudes necessary to develop healthy identities, manage their emotions, set and achieve positive personal and collective goals, feel and show empathy for others, establish and maintain positive and supportive interpersonal relationships, manage interpersonal situations in a constructive way and make responsible and caring decisions (Payton et al., 2008; Durlak et al., 2011; Gutman and Schoon, 2013; Collaborative for Academic, Social, and Emotional Learning, 2015, 2020; Weissberg et al., 2015; Oberle et al., 2016; Taylor et al., 2017; Jagers et al., 2019; Mahoney et al., 2020).

SEL programmes promote the development of a series of socio-emotional competencies in the participating students, in addition to promoting certain changes in their immediate environment and surroundings, with the aim of generating substantial improvements in their well-being and performance (Diekstra, 2008; Payton et al., 2008; Durlak et al., 2010, 2011; Sklad et al., 2012; Collaborative for Academic, Social, and Emotional Learning, 2015, 2020; Jagers et al., 2015, 2019; Wigelsworth et al., 2016; Jones and Kahn, 2017; Sabey et al., 2017; Taylor et al., 2017; Corcoran et al., 2018; Aguilar et al., 2019; National Commission on Social, Emotional, and Academic Development, 2019; Yang et al., 2019; Mahoney et al., 2020; Murano et al., 2020). Specifically, these variables were defined in the following terms: (a) Self-awareness or skills to recognize one's own emotions, thoughts and values, strengths, and limitations, as well as how they influence behavior in different situations, while fostering an adequate self-perception (e.g., self-concept, self-esteem, self-efficacy) and confidence in one's own abilities (e.g., growth mindset) (Collaborative for Academic, Social, and Emotional Learning, 2015, 2020; Jagers et al., 2019; Panorama Education, 2020); (b) Social awareness or cognitive ability that includes skills to develop empathy, perspective-taking, appreciation of diversity and respect for others, regardless of their origin or characteristics, respecting social norms and understanding the influence of the immediate environment (e.g., family, school, community) (Collaborative for Academic, Social, and Emotional Learning, 2015, 2020; Jagers et al., 2019; Panorama Education, 2020); (c) Self-control or the ability to manage one's impulses and emotions effectively in different situations, handling stress appropriately, delaying gratification, including motivation (e.g., resilience, will, discipline, perseverance, organizational, and planning strategies) to achieve personal and collective goals and objectives (Collaborative for Academic, Social, and Emotional Learning, 2015, 2020; Jagers et al., 2019; Panorama Education, 2020); (d) Relationship skills or the ability to establish and maintain healthy, supportive relationships and interact effectively with others (e.g., active listening, empathetic communication, seeking and offering help, conflict resolution, resisting negative social pressure, and working in groups) (Collaborative for Academic, Social, and Emotional Learning, 2015, 2020; Jagers et al., 2019; Panorama Education, 2020); (e) Responsible decision-making or the ability to consider ethical, safety and social factors when making decisions, so that the individual is able to deal responsibly with academic and social situations in everyday life and contribute to the well-being of the community (Collaborative for Academic, Social, and Emotional Learning, 2015, 2020; Jagers et al., 2019); (f) School climate or perception of the social and learning climate of the school and classroom, i.e., the quantity and quality of interactions with other members of the educational community (Thapa et al., 2013; Panorama Education, 2020); (g) Sense of belonging or feeling of belonging to the school community and feeling an important member of it (Panorama Education, 2020); (h) School safety or perceptions of physical and psychological safety in the school and classroom (Panorama Education, 2020); (i) Well-being or the degree to which a person is fully functioning physically, mentally, and socially, associated with the realization of one's true potential (Ryan and Deci, 2001; Panorama Education, 2020); and (j) Academic performance or results obtained throughout the training process until the corresponding qualification is obtained, i.e., overcoming the minimum requirements or objectives established for passing a subject, subject, course, cycle, or qualification (Rivkin et al., 2005; González et al., 2019).

Obviously, the measurement of the dependent variables had to be done in quantitative terms, using standardized tests, tests, questionnaires, inventories, scales, or structured interviews. The methodological designs adopted by the selected studies were experimental and quasi-experimental designs with comparison groups (Campbell and Stanley, 1963). Logically, in order to set up the groups, it was essential that they used random assignment or matching with appropriate adjustments for any differences in the pretest phase.

In terms of the participating population, studies were limited to children in early childhood, primary and secondary education (i.e., ages 3–18), as these are the educational stages in which this type of programme is most developed and recommended, especially as a preventive measure against the problems of school adjustment that students tend to present (Taylor et al., 2017; Jones et al., 2019; Mahoney et al., 2020).

Finally, geographical and/or cultural restrictions were established in this systematic review, limiting the selection of studies to those carried out in Latin America, mainly because the available meta-analyses on SEL programmes have focused mainly on research carried out in the Anglo-Saxon context, or those that include cross-cultural adaptations originally validated in this context (Wigelsworth et al., 2016). It was also decided that the language of publication of the studies to be included in this research work would be English and Spanish, and no time restriction was applied.

### Search Strategy

The literature search was carried out through various procedures and resources in order to ensure the inclusion of all studies related to the subject matter of this research, whether published or unpublished. To do this, firstly, a primary search was carried out on the available electronic platforms and databases, while secondly, a complementary search was carried out, accessing other resources and websites of relevant networks and institutions, contacting experts, and carrying out manual searches, among other actions. The search was conducted in February 2021.

The electronic platforms and databases selected for the primary search were Web of Science (Science Citation Index Expanded; Social Science Citation Index; Arts and Humanities Citation Index; Conference Proceedings Citation Index-Science; Conference Proceedings Citation Index-Social Science and Humanities; Book Citation Index-Science; Book Citation Index-Social Sciences and Humanities; Current Chemical Reactions; Index Chemicus; Emerging Sources Citation Index; BIOSIS Citation Index; BIOSIS previews; Current Contents Connect; Derwent Innovations Index; Korean Journal Database; MEDLINE; Russian Science Citation Index; SciELO Citation Index), ProQuest (ABI/INFORM Collection; APA PsicoArticles® APA PsicoExtra® APA PsicoInfo® APA PsicoTest® Arts and Humanities Database; Coronavirus Research Database; Early Modern Books; E-book Central; EconLit; Entrepreneurship Database; Health and Medical Collection; MEDLINE; Nursing and Allied Health Database; Periodicals Archive Online; Periodicals Index Online; ProQuest Dissertations and Theses Global; Psychology Database; Social Science Premium Collection; Education Collection; International Bibliography of the Social Sciences; Library and Information Science Collection; Social Science Database; Sociology Collection) and Scopus.

The complementary search involved the following actions: (a) Manual searches of the reference lists of each of the studies included in this systematic review; (b) Google Scholar searches, aimed at identifying unpublished studies on the web; (c) personal contacts with national and international researchers of recognized prestige, with the aim of identifying unpublished reports and research in development or in progress; (d) searches in open access resources or gray literature: OpenGrey GreyNet International-Gray Literature Network Service, National Technical Information Service (NTIS), Directory of Open Access Repositories (OpenDOAR), Open Access Scholarly Information Sourcebook (OASIS), Bielefeld Academic Search Engine (BASE), COnnecting REpositories (CORE) y Library Hub Discover; (e) resource searches on development research: Community Research and Development Information Service (CORDIS); Economic and Social Research Council (ESRC) Regard database, Center for Reviews and Dissemination (CRD), NBER Working Papers, The Campbell Collaboration and RePEc; and (f) searches in relevant networks and institutions: What Works Clearinghouse, Evidence for ESSA, EPPI Center, Educational Evidence Portal (EPP), IZA World of Labor, Social Science Research Network (SSRN), The Campbell Collaboration, CASEL and Panorama Education.

Search terms were selected using the Education Resources Information Center Thesaurus, based on the study inclusion criteria specified above, trying to strike a balance between sensitivity (i.e., identifying all articles on the topic) and specificity (i.e., identifying only relevant articles).

The search strategy was modified according to the specifications of each platform and electronic database, as well as any other resources used (e.g., Google Scholar). In this sense, for those resources, platforms and electronic databases with advanced search functions, the search terms (in English and Spanish) were classified into two categories (i.e., independent variable and dependent variables, excluding terms related to methodology, population and geographical or cultural restrictions to ensure sensitivity and specificity), which were included in the various search engines to identify the papers under study from the title, abstract and keywords. These categories were combined using the Boolean operator “AND,” while the Boolean operator “OR” was used for the search terms in each category and their synonyms. For resources or databases with basic search functions, search terms were adjusted to the limited functionality of their search engines, so searches were conducted by keyword and/or topic-topic, combining or including separate search terms. For example, the terms and combinations used in the search of Web of Science, ProQuest, and Scopus are specified below: (“social and emotional learning” OR “social and emotional aspects of learning” OR “socioemotional learning” OR “socio-emotional learning”) AND (“intervention^*^” OR “program^*^” OR “practice^*^” OR “train^*^” OR “initiative^*^” OR “action^*^” OR “project^*^”) AND (“competence^*^” OR “self-awareness” OR “self-perception^*^” OR “self-efficacy” OR “self-concept” OR “self-steam” OR “growth mindset” OR “social awareness” OR “empathy” OR “social perspective-taking” OR “self-management” OR “self-control” OR “emotion^*^” OR “feeling^*^” OR “attitude^*^” OR “behavio^*^” OR “stress management” OR “motivation” OR “self-discipline” OR “perseverance” OR “grit” OR “learning strateg^*^” OR “metacognit^*^” OR “resilience” OR “relationship skills” OR “social skills” OR “problem solving” OR “problem-solving” OR “resolving conflict^*^” OR “conflict resolution” OR “coping” OR “teamwork^*^” OR “leadership” OR “responsible decision-making” OR “school climate” OR “social climate” OR “classroom climate” OR “students environments” OR “educational environment” OR “classroom environment” OR “sense of belonging” OR “engagement” OR “school safety” OR “well-being” OR “welfare” OR “satisfaction” OR “success” OR “performance” OR “failure” OR “achievement” OR “grade point average” OR “GPA” OR “retention” OR “repetition” OR “dropout” OR “graduation”).

On the other hand, Refworks was used for the management and documentation of the process, as it allows the tracking of each of the studies identified in the search process. Therefore, the bibliographic information of the studies resulting from the searches was imported into Refworks and, in order to maximize the transparency and replicability of the search process, the team stored and still has the records, which include the database interface, the type of database, the customized search strategy, the search terms and language, the number of records obtained, search dates and the researcher's initials.

The selection process of the identified studies was carried out by implementing the following actions: (a) first level of screening, where two team members worked in parallel to identify and eliminate duplicate records; (b) second level of screening, where two team members identified and eliminated in parallel those studies that, after careful examination of their title and abstract, did not clearly meet the inclusion criteria; and (c) third level of screening, where two team members in parallel read the full text versions of the studies to determine their eligibility against the inclusion criteria. At the second and third levels, an *ad hoc* selection template was used, while the procedure followed to resolve discrepancies between task force members consisted of an additional review of the full text of the study and discussion of compliance with the inclusion criteria, mediated by a third task force member.

Once the final sample of studies had been confirmed, two members of the team extracted and coded the data and information from each study in parallel around the following variables (Lipsey and Wilson, 2001): (a) contextual characteristics (i.e., reference, country and type of publication); (b) methodological characteristics (i.e., sample selection procedure and group configuration, methodological design, data analysis and biases); (c) sample characteristics (i.e., size, age, gender, stage and educational level); (d) assessment instruments (i.e., standardized tests, tests, questionnaires, inventories, scales or structured interviews used to measure the dependent variables); (e) characteristics of the independent variable or SEL programmes (i.e., the environment in which it is developed, areas or components of the programme, duration of programme, and procedures, practices, strategies, techniques and/or resources of programme); (f) dependent variables (i.e., self-awareness, social awareness, self-control, relationship skills, responsible decision-making, school climate, sense of belonging, school safety, well-being, and academic achievement); and (g) results and conclusions.

This extraction and coding work has been carried out in a data extraction sheet (Excel), while disagreements about it have been resolved through consultation and discussion with a third member of the working team. Finally, the approach adopted for data analysis was, considering the aim of this systematic review, a narrative content analysis (Dochy, 2006).

## Results

**Figure 1:**
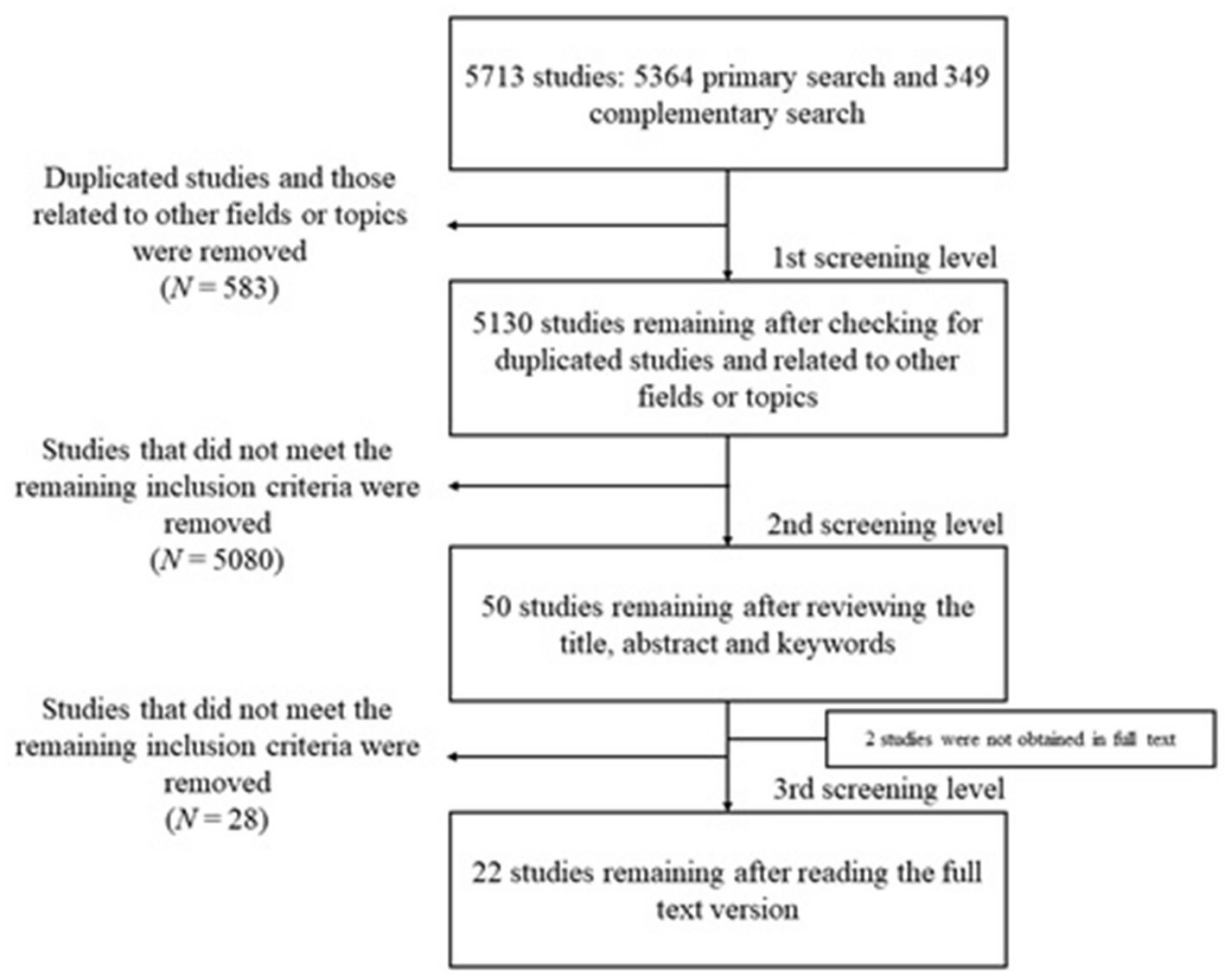
PRISMA flow chart for the literature search and screening (Page et al., 2021).

The general process of searching and selecting records was represented graphically in a PRISMA flow chart (Figure 1) (Page et al., 2021).

The selected studies come from four countries: 12 from Portugal, six from Spain, two from Brazil and two from Chile. The studies were published between 2010 and 2020 (2010 = 1, 2012 = 1, 2013 = 2, 2014 = 2, 2015 = 1, 2016 = 4, 2017 = 3, 2018 = 3, 2019 = 1, 2020 = 4). The language of publication was English (*N* = 18), Portuguese (*N* = 2), and Spanish (*N* = 2). Of the studies reviewed, all 23 were journal articles.

In terms of the total sample, 17,104 participants were examined in these studies (*M* = 777.45). The sample size ranged from 50 participants to 5,145 participants. The gender distribution did not vary between the studies, all 23 included samples composed of both males and females. The ages of the participants ranged from 5 to 17 years. The educational stage on which the studies focused was primary education (*N* = 13) and secondary education (*N* = 9).

The sampling techniques were distributed as follows: 19 studies used non-probability sampling and three used probability sampling. Regarding cluster configuration, 19 studies used non-random clustering and three used random clustering. Finally, reporting bias was included in 21 studies (95.45%) and missing in 1 study (4.55%).

In relation to the assessment instruments, 21 studies used standardized scales and only one study used *ad hoc* instruments. Regarding the research design, all 22 studies included a quasi-experimental pretest-post-test design, all of them including a non-equivalent control group design.

In terms of research context, the vast majority of experiences took place in public schools located in urban settings (19 out of the total number of papers). However, there are studies that covered the implementation of SEL programmes from an urban and rural perspective (three with a comparative character).

Regarding the characteristics of the programmes, Table 1 shows the main strategies and techniques that were used during their development. It shows that the duration of the programmes varies, with the longest lasting a total of 4 years (Moreira et al., 2010), and the shortest of 1 month (Berger et al., 2014). As can be seen, a wide variety of techniques are used to promote socioemotional competences, such as role-playing, assemblies, mindfulness, musical dynamics, storytelling, or dramatization, among others.

**Table 1:** Most relevant characteristics of the SEL programmes implemented.

**Reference**	**Components**	**Duration**	**Procedure**	**Instrument**
Santos et al. (2020a)	Relationship management; decision making; self-management; social consciousness	7 months	Interspersed outdoor/indoor classroom sessions using debriefing as a technique.	TEC; EQi:YV; Eqi; PCIS; PACS
Cejudo et al. (2020)	Assertiveness; Self-esteem; Decision-making; Emotional Intelligence; Addictions; Conflict Management	1 year	Development of socio-emotional skills through the video game “Isolated.”	KIDSCREEN; SWLS; PANAS; MH-5; TEIQue-ASF
Mira-Galvañ and Gilar-Corbi (2020)	Emotional regulation	6 months	Each class incorporated 10–15 min for assembly, ~5 min for mindfulness practice and 30 min per week (3 times a month) to work on different activities related to the programme.	EQi:YV
Santos et al. (2020b)	Cooperative learning; emotional intelligence; school climate	9 weeks	The programme was developed using 16 lessons from the book “I Can Problem Solve” (ICPS) which focuses on using social dynamics to encourage problem solving.	CPM; School Achievement Test; SSRS-BR
Luna et al. (2019)	Self-control, Relationship management; school climate; wellbeing and academic achievement	6 weeks	Work on socio-emotional skills through an alternative sport called “ringo.”	The Kidscreen-10 Index; The Positive and Negative Affect Schedule; TEIQue-ASF; SAS-A
Faria et al. (2018)	Self-Awareness; Emotional Regulation and Intelligence	7 months	Sessions using stories to work on different emotions. Mascots were created to accompany the students during the programme.	TEC
Coelho and Sousa (2018)	Self-awareness; social awareness and self-control	2 years	Development of emotional states through story characters. Narratives with open endings. Creating a wheel of emotions	QACSE; SDQ II
Rodríguez-Ledo et al. (2018)	Social-emotional competences; empathy; social awareness and social skills	9 months	Socio-emotional intelligence model in which they were divided into three key groups that worked on the following aspects: attention and understanding of emotions, emotional regulation, and social adjustment.	EDQ-SEC; EQi-YV; BAS3; IECA
Coelho and Sousa (2017a)	Self-awareness; social awareness; self-control	2 years	It includes all pupils in each class and is infused into the school curriculum and integrated into a multi-year project.	BAS-3; Global Self-Esteem scale; Self-Description Questionnaire II; QACSE-P
Pereira and Marques-Pinto (2017)	Self-awareness; Social awareness; Self-control; Relationship management; Decision-making; Sense of belonging; Well-being	12 weeks	The SEL programme uses an alternative approach using Education through Art. Musical dynamics as well as simple dances were used to work on these skills, as well as motor skills.	ESCQ; SSBS 2; MHC-SF
Coelho and Sousa (2017b)	Self-awareness; Social awareness; Self-control; Relationship management; Decision-making; Sense of belonging; Well-being	8 months	It includes all pupils in each class and is infused into the school curriculum and integrated into a multi-year project.	BAS-3; SDQ I
Coelho et al. (2016a)	Social awareness; self-control; social isolation; social anxiety; leadership; leadership; self-esteem	2 years	It includes all pupils in each class and is infused into the school curriculum and integrated into a multi-year project.	AF-5
Coelho et al. (2016b)	Social awareness; self-control; social isolation; social anxiety; leadership; leadership; self-esteem	1 year	The programme consists of four modules: (1) two sessions on self-awareness; (2) three sessions on developing self-management and social awareness; (3) three sessions on increasing self-esteem; and (4) two sessions on developing responsible decision-making.	BAS-3; SDQ I
Waldemar et al. (2016)	Social, academic, emotional, family, physical, and social self-concept	12 weeks	Breathing mindfulness exercises, reflective and playful activities as well as informal mindfulness exercises were set up.	Strengths and Difficulties Questionnaire; the Youth Quality of Life Instrument; the Swanson, Nolan and Pelham–IV questionnaire
Correia and Marques-Pinto (2016)	Social, academic, emotional, family, physical, and social self-concept	18 weeks	Activities in the “Salto de Gigante” programme included showing instructional videos, storytelling, educational group games, role-playing and artistic expression activities, brainstorming strategies, modeling, constructive feedback, individual positive reinforcement, and group discussion/reflection.	ACES; SSBS-2; BERS-2
Coelho et al. (2015)	Hyperactivity; Emotional problems; Behavioral problems; Behavioral problems	2 years	It includes all pupils in each class and is infused into the school curriculum and integrated into a multi-year project.	BAS-3; SDQ I
Coelho et al. (2014)	School Climate; Academic Behavior; Social Competences; Self-awareness	2 years	It includes all pupils in each class and is infused into the school curriculum and integrated into a multi-year project.	AFA
Berger et al. (2014)	Social awareness; self-control; social isolation; social anxiety; leadership; leadership; self-esteem	1 month	The BASE programme is based on four pillars: (a) holistic developmental perspective; (b) systemic-interactional approach with linkages as a central aspect; (c) students' developmental processes and the construction of a positive self-narrative; and (d) the importance of professional teacher training.	Self-report scale of socio-emotional wellbeing; social climate scale; TAE; Social Cognitive Mapping
Milicic et al. (2013)	Academic, social, emotional, and family self-concept	7 months	The modality of the programme is that of a workshop in which the aim is to generate a space for conversation, personal and collective reflection, and the exercise of socio-emotional competencies in a space of affective support.	Self-report scale of socio-emotional wellbeing; Social climate scale; TAE; Social Cognitive Mapping
Castillo et al. (2013)	Social-emotional well-being; self-esteem; school climate	2 years	Different emotions were worked on through different role plays, art projects, film forums and reflection activities.	AQ and IRI Spanish version
Pérez-Escoda et al. (2012)	Emotional intelligence	9 months	Exercises were carried out to recognize the emotions of others, with conflict resolution techniques, role-playing exercises, debates, and group dynamics.	EQ-i: YV
Moreira et al. (2010)	Self-control; Emotional differentiation; Emotional regulation; Emotional control	4 years	The exploration of socio-emotional concepts was combined with the learning of curricular subjects such as civics, Portuguese language, environment, and mathematics.	CSCS; SPPC; EII; CABS; ERCSI

The instruments used to measure the different constructs assessed in the experiences are also detailed. The Test of Emotion Comprehension (TEC), the Global Self-Esteem scale and the Socialization Battery 3 (BAS-3) stand out for their frequency of use.

On the other hand, Table 2 shows the distribution of the studies when grouped by analyzed outcomes of the variables linked to the SEL model. For the variable “self-awareness,” a total of four studies (18.18%) were found, three of them obtained significant differences between groups and only one showed no difference. Regarding “social awareness,” a total of seven studies (31.82%) were found, of which six reported significant differences. Regarding “self-control,” nine studies (40.91%) were found, eight reported significant differences between the groups and only one reported no differences. In “relationship skills,” eight studies (36.36%) were collected, five with significant differences and three that reported no differences between groups. In “decision-making,” two studies were found (9.09%), of which only one showed significant differences. In “school climate,” four studies (18.18%) were found, all four with significant differences. In “well-being,” eight studies (36.36%) were found, six with significant differences and two with no differences. And finally, in “academic performance,” six studies (27.27%) were found, three with significant differences and three that reported no significant differences. There were no studies that collected the variables SEL: sense of belonging or school safety.

**Table 2:** Study results for each dependent variable of the SEL model.

**Outcomes**	**Statistically not significant results**	**Statistically significant results**
Self-awareness	Rodríguez-Ledo et al. (2018)	Coelho et al. (2016a); Faria et al. (2018); Santos et al. (2020a)
Social awareness	Coelho and Sousa (2017a)	Coelho et al. (2014, 2015, 2016b); Coelho and Sousa (2017b, 2018); Santos et al. (2020a)
Self-control	Coelho and Sousa (2017a)	Moreira et al. (2010); Coelho et al. (2015, 2016b); Coelho and Sousa (2017b, 2018); Pereira and Marques-Pinto (2017); Santos et al. (2020a,b)
Relationship skills	Castillo et al. (2013); Rodríguez-Ledo et al. (2018); Santos et al. (2020a)	Pérez-Escoda et al. (2012); Correia and Marques-Pinto (2016); Waldemar et al. (2016); Pereira and Marques-Pinto (2017); Coelho and Sousa (2018)
Decision-making	Coelho and Sousa (2018)	Santos et al. (2020a)
School climate		Milicic et al. (2013); Berger et al. (2014); Correia and Marques-Pinto (2016); Waldemar et al. (2016)
Well-being	Pereira and Marques-Pinto (2017); Rodríguez-Ledo et al. (2018)	Pérez-Escoda et al. (2012); Milicic et al. (2013); Berger et al. (2014); Coelho et al. (2014); Luna et al. (2019); Cejudo et al. (2020)
Academic performance	Coelho et al. (2014); Pereira and Marques-Pinto (2017); Santos et al. (2020a)	Berger et al. (2014); Correia and Marques-Pinto (2016); Mira-Galvañ and Gilar-Corbi (2020)

## Discussion and Conclusions

The purpose of this systematic review was to synthesize the main characteristics and evidence on the effectiveness of SEL programmes to improve school and socio-emotional outcomes of early childhood, primary and secondary education pupils in Ibero-American contexts. In this regard, with respect to the first research question, the results obtained show that Portugal, Spain, Chile, and Brazil have been the Ibero-American countries that have made the greatest commitment over the last decade to the application and, especially, the evaluation of this type of interventions in compulsory education (i.e., primary and secondary education). Particularly, by a systematic and rigorous evaluation, characterized by the selection of quasi-experimental evaluation methodological designs, with comparison groups, mostly non-randomly configured, but with statistical adjustments that guaranteed their equivalence in the pretest phase. In the rest of the Ibero-American countries, as in the early childhood education stage, no studies with these characteristics have been identified regarding SEL practices.

In fact, the number of programmes incorporated in this review can be considered quite small, fundamentally when compared to the number of interventions included in systematic reviews developed in the Anglo-Saxon field (e.g., Diekstra, 2008; Payton et al., 2008; Durlak et al., 2010, 2011; Sklad et al., 2012; Jagers et al., 2015; Wigelsworth et al., 2016; Sabey et al., 2017; Taylor et al., 2017; Corcoran et al., 2018; Yang et al., 2019; Murano et al., 2020). However, these results are determined by the large number of SEL experiences that have been excluded throughout the selection process of this work, mainly for one of the following reasons: (a) they did not incorporate evaluation measures, or provided exclusively participation and/or satisfaction results among their participants; (b) they employed qualitative or pre-experimental evaluation methodological designs; or (c) they incorporated quasi-experimental designs, with comparison groups, but with quite limited intergroup comparability.

Regarding the most relevant characteristics of the SEL programmes included in this systematic review, the second research question, the results show that, in general terms, most of these interventions have been developed in urban educational centers, during school time, being implemented by teachers and/or external personnel, often trained for this purpose, as part of the curriculum or tutorial action. In addition, they have a variable duration of more than 12 sessions, with a weekly or biweekly frequency, over a minimum of 5 months, incorporating a wide variety of procedures, practices, strategies, techniques and/or resources (e.g., games, role-playing, video games, forums, debates, stories, art projects, alternative sports, individual, pair and group reflections, dance, mindfulness practices, personal counseling, direct instruction, modeling and group dynamics). However, all SEL programmes, as established in the specialized literature (Durlak et al., 2010, 2011), are mainly aimed at enhancing the development of self-awareness, social awareness, self-control, relationship skills and responsible decision making, using a step-by-step sequenced training approach that emphasizes active forms of learning, concentrating specific time and attention on skills training and in which goals are clearly defined, i.e., sequenced, active, focused and explicit training. Likewise, it is inevitable to highlight the number of studies that have been conducted on some of these SEL interventions, as is the case of the “Bienestar y Aprendizaje Socioemocional (BASE)” programme (Milicic et al., 2013; Berger et al., 2014), but especially the high number of editions that have been carried out of the “Actitud positiva en la escuela” SEL programme, not to mention its large-scale implementation in different geographical areas of Portugal (Coelho et al., 2014, 2015, 2016a,b; Coelho and Sousa, 2017a,b, 2018).

Regarding the third research question, in order to identify evidence on the effectiveness of SEL programmes developed in the Ibero-American context, the results of this systematic review, coming from the impact evaluation of these interventions, are very similar to the results provided by the available systematic reviews on SEL experiences in the Anglo-Saxon context (e.g., Diekstra, 2008; Payton et al., 2008; Durlak et al., 2010, 2011; Sklad et al., 2012; Jagers et al., 2015; Wigelsworth et al., 2016; Sabey et al., 2017; Taylor et al., 2017; Corcoran et al., 2018; Yang et al., 2019; Murano et al., 2020). Therefore, it can be stated that the SEL programmes included in this work also generate improvements among the participating students at the socioemotional level, which usually translates into greater well-being and school performance (Collaborative for Academic, Social, and Emotional Learning, 2015, 2020; Jones and Kahn, 2017; Aguilar et al., 2019; Jagers et al., 2019; National Commission on Social, Emotional, and Academic Development, 2019; Mahoney et al., 2020), although it is true that the percentage of these interventions that include among their dependent variables certain socioemotional competencies (i.e., responsible decision-making), changes in the most immediate environment (i.e., school climate, school safety, and sense of belonging) or indicators of school well-being and performance was quite low, below 35%. However, despite this, as stated by Slavin (2016), the power of the available evidence on the SEL programmes in this systematic review can be considered “moderate,” as they are supported by at least one quasi-experimental study, allowing them to qualify as evidence-based practices.

On the other hand, if we analyse the relationship between the main characteristics of the SEL programmes included in this systematic review and their effectiveness, we can see that the interventions that have been developed in out-of-school time (e.g., Santos et al., 2013; Salgado and Marques-Pinto, 2017), that employ video games (e.g., Cejudo et al., 2020) or alternative sports (e.g., Luna et al., 2019) among their procedures, practices, strategies, techniques and/or resources, with exclusively compulsory secondary education population (e.g., Castillo et al., 2013; Rodríguez-Ledo et al., 2018; Luna et al., 2019; Cejudo et al., 2020), are associated to a greater extent with a lower impact on the dependent variables considered, yielding results that are not statistically significant. Quite the opposite happens with those SEL programmes implemented in school time, as part of the curriculum, in which teachers and/or responsible external staff are trained to carry out a sequenced, active, focused, and explicit SEL training with the participating students (e.g., Milicic et al., 2013; Coelho et al., 2014, 2015, 2016a,b; Berger et al., 2014; Waldemar et al., 2016; Coelho and Sousa, 2017a,b; Coelho and Sousa, 2018; Mira-Galvañ and Gilar-Corbi, 2020), as they reveal a greater impact on socioemotional competencies, well-being, and school achievement.

These results align with many of the key indicators that ensure the success of SEL programmes (e.g., explicit SEL instruction, SEL integrated with school instruction, active role of participants, training of staff responsible for implementation) (Collaborative for Academic, Social, and Emotional Learning, 2020; Mahoney et al., 2020). However, other key indicators related to collaboration and synergies between classrooms, schools, families, and communities, as well as the inclusion of continuous improvement systems (Collaborative for Academic, Social, and Emotional Learning, 2020; Mahoney et al., 2020), are underrepresented in the SEL experiences that have been developed in Ibero-America. It is also necessary to highlight the high frequency with which personnel external to the educational centers are employed for the implementation of these programmes, which considerably affects their sustainability, in addition to the need to further strengthen the fidelity of implementation and the quality of the methodological evaluation designs.

Obviously, this systematic review is not without limitations: (a) the literature search, both primary and complementary, was completed in February 2021, so literature published after that date has not been included in the systematic review; (b) researcher bias in selecting manuscripts may have influenced the final sample of studies, although its effects were attempted to be attenuated by the parallel development of the selection process by two researchers, resolving any dissonance with the participation of a third investigator; and (c) the amount of gray literature included in the review is limited, despite the use of the different search engines that collect this type of literature, which may have increased the threat of publication bias, although a large number of these studies were actually excluded in the selection process, mainly because they used low-quality methodological designs.

As future lines of research, a meta-analysis of the studies collected should be carried out in order to find out the overall effect size of the research. It is also worth highlighting the possibility of continuing to implement SEL programmes in the Ibero-American context with the variables sense of belonging or school safety, since at present no study addresses these SEL variables. The practical implications of the work are linked to the establishment of the key implementation factors of the SEL models for each variable, in terms of programme components, duration and integration. This shows scientific evidence that can ensure the success of future programmes.

The scientific production on the design, implementation, and evaluation of SEL programmes in the Ibero-American context over the last decade differs greatly from the research, practices and policies that have been developed over the last 25 years in the Anglo-Saxon context (e.g., National Commission on Social, Emotional, and Academic Development, 2019), both in quantity and quality. In this sense, the need to improve research on this type of interventions in Ibero-American countries seems evident, in addition to establishing national and international agendas that promote their adoption throughout the educational system, placing them at the center of education, along with school performance, so that they are coordinated and integrated with existing educational priorities (Collaborative for Academic, Social, and Emotional Learning, 2020). Of course, this requires ongoing commitments and efforts on the part of all the agents involved, without forgetting that these efforts must be adequately resourced if the aim is for children and young people participating in these programs to reach their full potential (Mahoney et al., 2020).

## Data Availability Statement

The original contributions presented in the study are included in the article/supplementary files, further inquiries can be directed to the corresponding author/s.

## Author Contributions

J-MR-R and GG-G collected and analyzed the data. F-DF-M, J-MR-R, J-AM-M, and GG-G assisted in literature review and wrote the initial draft of the manuscript. F-DF-M and J-AM-M monitored and supervised all aspects of the study. All authors approved the final version of the paper.

## Funding

This work has been funded by Porticus and the Tomillo Foundation, through the R+D+i Project entitled Evaluation of the Itinerario + Educational Model – Phase I, established between the Office for the Transfer of Research Results (OTRI) of the University of Granada and the Tomillo Foundation (Reference: CNT4547).

## Conflict of Interest

The authors declare that the research was conducted in the absence of any commercial or financial relationships that could be construed as a potential conflict of interest.

## Publisher's Note

All claims expressed in this article are solely those of the authors and do not necessarily represent those of their affiliated organizations, or those of the publisher, the editors and the reviewers. Any product that may be evaluated in this article, or claim that may be made by its manufacturer, is not guaranteed or endorsed by the publisher.
